# CORRIGENDUM

**Published:** 1988


					CORRIGENDUM
ACKNOWLEDGEMENT
Papers
i) Varma, V.K., Chaturvedi, S.K., Malhotra A. and Chari. P (1983) Psychiatric aspects
of chronic intractable pain.
Indian Journal of Psychiatry, 25: 173-179
ii) Varma V.K., Malhotra, A. and Chaturvedi S.K. (1986) Socio-demographic study of
patients with chronic pain. Indian Journal of Psychiatry, 28: 119-125
Earlier published in the Journal were based on the Task Force study of the Indian
Council of Medical Research entitled "Illness behaviour in patients presenting with
pain".
Financial assistance provided by the I.C.M.R. for undertaking the above Task
Force study is gratefully acknowledged. Thanks are also due to the consultants of the
study, and ICMR staff of the Division of Noncommunicable Diseases.
The above acknowledgement was missed in the above published papers. The
omission is sincerely regretted.
Vijoy K. Varma
Principal Investigator
The following references were inadvertantly not printed in the list of references for the ar-
ticle:
A. Venkoba Rao and S. Parvathi Devi. (1987), Psychobiology of Suicide Behaviour, Indian
Journal of Psychiatry, 29(4), 299-306.
References:
VENKOBA RAO, PARVATHI DEVI and SRINIVASAN, V. (1984) Low melatonin
Syndrome of depression. Proceeding of the 17th South Zone Annual Conference of the
Indian Psychiatric Society, Coimbatore, October, 1984.
VENKOBA RAO, A. (1986) Depressive Disease, Indian Council of Medical Research,
New Delhi, p.81.
The omission is sincerely regretted.
EDITOR

				

